# Wernicke’s Encephalopathy Secondary to Hyperemesis Gravidarum in Pregnancy: A Case Report

**DOI:** 10.1002/ccr3.72607

**Published:** 2026-04-24

**Authors:** Abhishek Mahato, Sameer Parajuli, Bandana Ghimire, Bishal Basnet, Bishal Sigdel, Bishal Kumar Yadav, Sagar Kumar Jha

**Affiliations:** ^1^ Institute of Medicine Tribhuvan University Nepal

**Keywords:** hyperemesis gravidarum, pregnancy, thiamine deficiency, Wernicke's encephalopathy

## Abstract

Wernicke's Encephalopathy, although classically associated with chronic alcoholism, can also occur during pregnancy as a complication of hyperemesis gravidarum and is often underdiagnosed. We report a case of a 34‐year‐old pregnant woman at 16 weeks of gestation who presented with prolonged vomiting, altered mental status, nystagmus, and gait ataxia. Magnetic Resonance Imaging of the brain revealed characteristic bilateral thalamic and periaqueductal hyperintensities. Pregnancy increases thiamine requirements, and persistent vomiting in hyperemesis gravidarum can rapidly precipitate thiamine deficiency, leading to Wernicke's Encephalopathy; however, the classical triad is frequently incomplete, contributing to delayed diagnosis. Early recognition supported by imaging findings is crucial, as prompt administration of intravenous thiamine resulted in significant neurological recovery in our patient. This case highlights the importance of maintaining a high index of suspicion for Wernicke's Encephalopathy in pregnant women presenting with hyperemesis gravidarum and neurological symptoms, as timely parenteral thiamine therapy can lead to favorable maternal and fetal outcomes.

## Key Clinical Message

Wernicke's Encephalopathy should be suspected in pregnant women with Hyperemesis Gravidarum who develop neurological symptoms, even if the classical triad is incomplete. Hyperemesis Gravidarum depletes thiamine stores in pregnancy; early empiric administration of parenteral thiamine, before imaging, is essential to prevent irreversible neurological damage and improve maternal and fetal outcomes.

## 1 Introduction

Wernicke's encephalopathy is an acute neuropsychiatric condition caused by thiamine (Vitamin B1) deficiency that requires immediate medical attention to avoid neurological morbidity and death [1]. Thiamine serves as a crucial coenzyme in the metabolism of carbohydrates, fats, and proteins, as well as in mitochondrial energy production and neurotransmitter synthesis [2]. The classical clinical triad of Wernicke's Encephalopathy includes an abnormal mental state, ataxia, and nystagmus, which is observed in merely 16%–33% of patients during the initial examination [3].

Wernicke's encephalopathy, while often linked to alcoholism, is frequently underrecognized in nonalcoholic individuals, and a delayed diagnosis can lead to unfavorable clinical results [4]. Indicators of nonalcoholic Wernicke's encephalopathy encompass conditions that lead to prolonged vomiting or chronic diarrhea, such as Hyperemesis Gravidarum, post‐bariatric surgery states, and inflammatory bowel disease [5]. About 80% of individuals suffering from untreated Wernicke's Encephalopathy (WE) progress to develop Korsakoff syndrome, a condition marked by memory deficits accompanied by confabulation [4].

Hyperemesis Gravidarum (HG), characterized by extreme nausea and vomiting during pregnancy, is regarded as one of the most feared complications of gestation, impacting approximately 1.5%–3.0% of expectant mothers [6]. Hyperemesis Gravidarum (HG) may continue during pregnancy, leading to malnutrition, dehydration, electrolyte imbalances, and unintentional weight loss, which often necessitates hospitalization [7]. Effective treatment of Hyperemesis Gravidarum requires a combination of medical interventions, lifestyle modifications, dietary adjustments, supportive care, and patient education [7]. This case report highlights an interesting clinical presentation of Wernicke's encephalopathy, aiming to raise awareness about the potential complications associated with Hyperemesis Gravidarum.

## 2 Case Presentation

A 34‐year‐old pregnant woman at the 16th week of gestation (G2P1L1) was admitted to the Emergency unit of our center with the chief complaints of multiple episodes of vomiting for the past 2 months and altered level of consciousness for 5 days prior to presentation. The vomiting had been occurring since the 1st month of pregnancy for which she was managed with dietary modifications and intravenous antiemetic therapy, including metoclopramide 10 mg as needed. Although vomiting had partially improved 2 weeks before presentation, the patient subsequently developed neuropsychiatric symptoms such as talking irrelevantly, failure to acknowledge relatives, and crying without any apparent reason. However, these episodes were intermittent, and at other times, she would have an intact level of consciousness. She also had difficulty walking on her own, but there was no history of fever, involuntary body movements, or dysarthria. The patient had a history of unintentional weight loss of approximately 5 kg. There was no history of alcohol abuse, chronic illness, or use of other medications aside from routine prenatal supplements.

On examination, the patient was conscious but intermittently disoriented to place, with impaired attention and short‐term memory. Her vital signs were stable. General systemic examination was unremarkable. On neurological examination, assessment of higher mental functions revealed disorientation to place, impaired attention and concentration, and difficulty in recent memory. Cranial nerve examination was largely intact; however, horizontal nystagmus was present. Motor system evaluation demonstrated normal muscle bulk and tone in all four limbs. Motor power was full (5/5) in the upper limbs, while the lower limbs showed mildly reduced strength (4/5) bilaterally, predominantly involving hip and knee flexion and extension. The observed bilateral lower‐extremity weakness (4/5) was documented as part of a routine neurological examination, and no specific clinical etiology was identified in this case. Additional coordination tests, including finger‐to‐nose and heel‐to‐shin, were performed and were within normal limits, with no evidence of cerebellar dysfunction apart from gait ataxia. Deep tendon reflexes were brisk in the upper limbs (++) and normal to mildly brisk (+) in the lower limbs. Plantar responses were bilaterally down‐going. Sensory examination was normal. Cerebellar assessment revealed gait ataxia with swaying of the body while standing and walking. There were no signs of meningeal irritation. The relevant laboratory parameters are summarized in Table 1.

**TABLE 1:** Laboratory Parameter of Patient.

Parameters	Tested value	Reference range
Liver function test		
Total serum bilirubin	10 mg/dL	0.3–1.2 mg/dL
Direct serum bilirubin	3 mg/dL	0–0.3 mg/dL
AST (Aspartate Aminotransferase)	44 U/L	5–40 (U/L)
ALT (Alanine Aminotransferase)	61 U/L	5–45 (U/L)
ALP (Alkaline Phosphatase)	51 U/L	40–120 U/L
Total protein	6.5 g/dL	6.0–8.0 g/dL
Complete blood count		
Hemoglobin	9.8 g%	13.5–18.0 g%
Red blood cell	3.16 million/μL	4.5–5.5 million/μL
PCV	27.7%	36%–54%
Total leukocyte count (TLC)	7800/cmm	4000–11,000/cmm
Renal function test		
Blood urea	1.44 mmol/L	1.6–7.0 mmol/L
Serum creatinine	36.09 μMol/L	60–130 μMol/L
Serum electrolyte		
Sodium	140 mEq/L	135–146 mEq/L
Potassium	4.3 mEq/L	3.5–5.2 mEq/L

## 3 Investigation

Other laboratory parameters, including alkaline phosphatase, total leukocyte count, platelet count, and differential counts were within normal limits.

Liver function tests show markedly elevated total serum bilirubin at 10 mg/dL (normal 0.3–1.2 mg/dL) and direct bilirubin at 3 mg/dL (normal 0–0.3 mg/dL), pointing to significant conjugated hyperbilirubinemia. The marked hyperbilirubinemia was likely related to cholelithiasis with biliary sludge causing cholestasis, although pregnancy‐related hepatic dysfunction may also have contributed. AST and ALT were mildly raised, suggesting mild hepatocellular involvement. Total protein is within range, but albumin is slightly low, which can occur in pregnancy due to hemodilution or mild liver stress. The complete blood count revealed anemia with normal red cell indices consistent with normocytic anemia, probably due to pregnancy‐related physiological changes combined with possible iron deficiency. Renal function tests displayed low blood urea and low serum creatinine, both of which are typical physiological findings in pregnancy due to increased glomerular filtration rate. Serum electrolyte levels were within the normal range.

**FIGURE 1:**
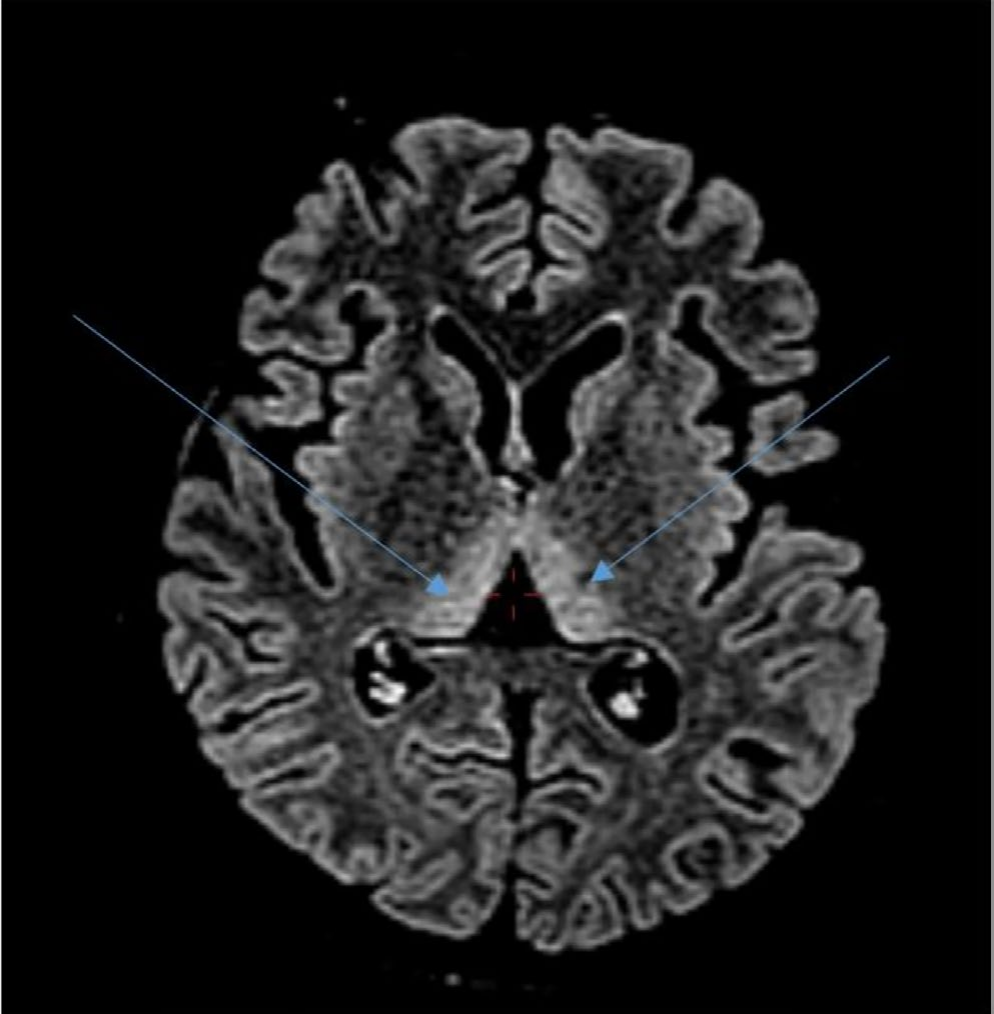
MRI of the brain (T2 sequence) showing high intensity signal in bilateral medial thalami.

**FIGURE 2:**
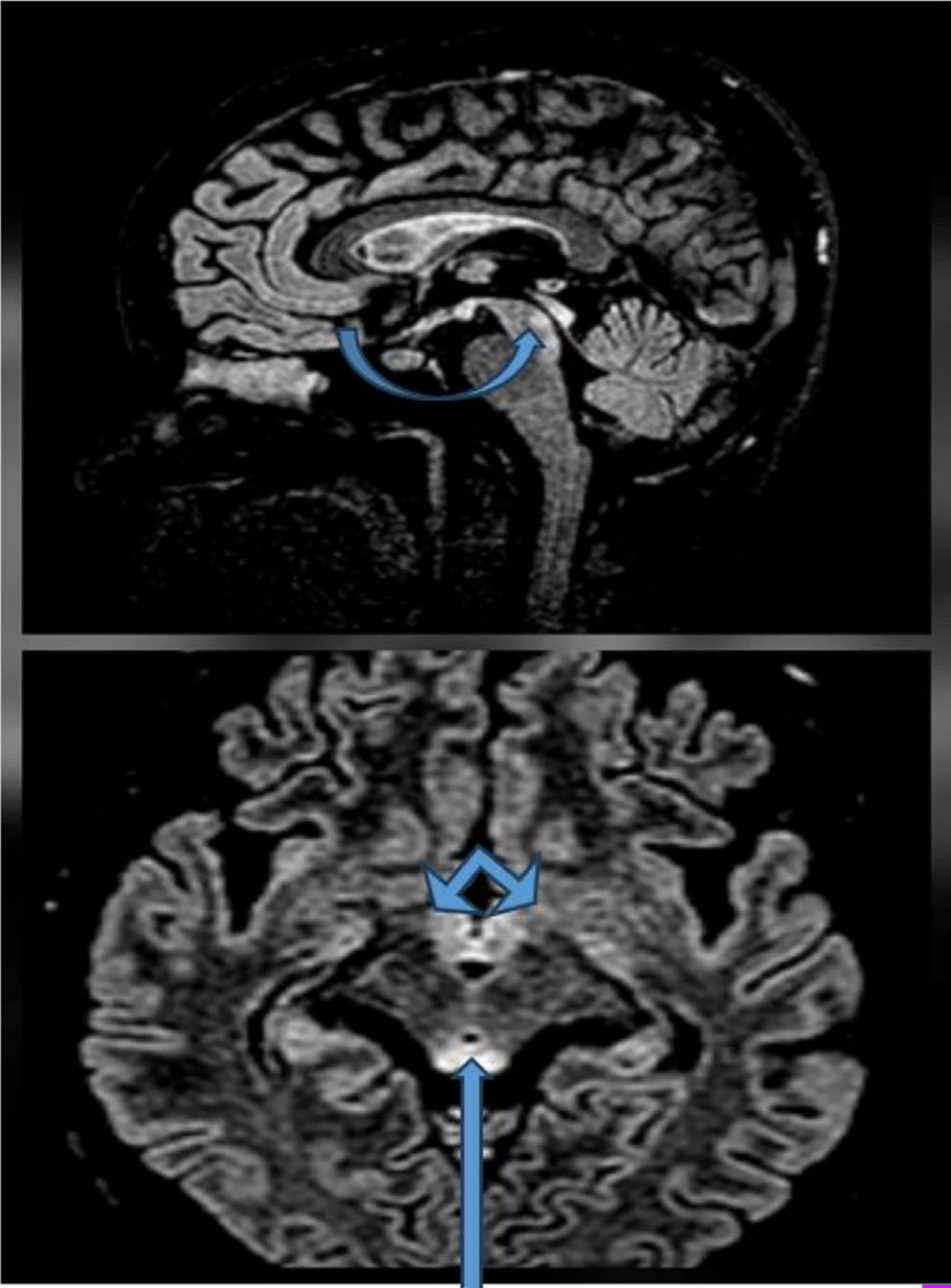
MRI of the brain (T2 sequence) showing high intensity signal in the periaqueductal gray matter and mammillary bodies.

**FIGURE 3:**
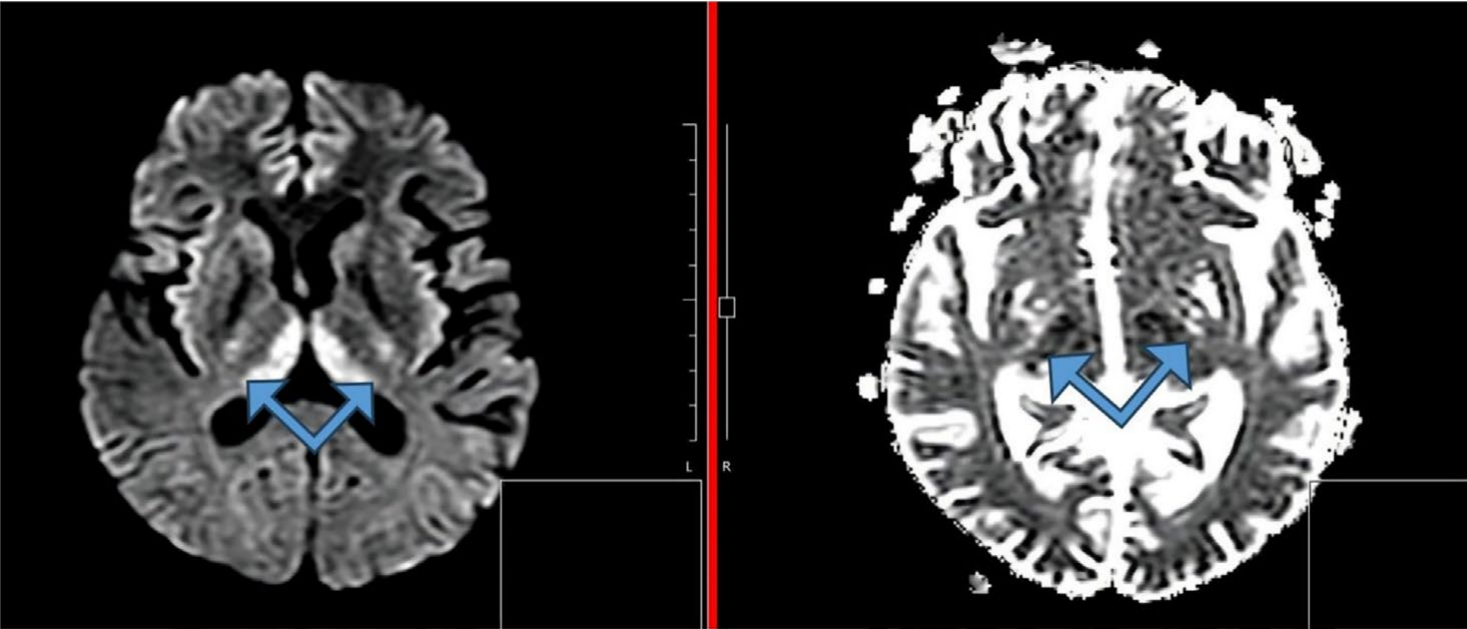
MRI of the brain showing diffusion restriction in the thalami.

The ultrasound of the abdomen and pelvis revealed cholelithiasis with biliary sludge, a gravid uterus, normal liver echotexture, and no retroplacental clot. Magnetic Resonance Imaging (MRI) of the brain showed T2/FLAIR high signal intensity in the bilateral medial thalami, periaqueductal gray matter, mammillary bodies, bilateral mesial temporal lobes, and insular cortex, findings suggestive of Wernicke encephalopathy (Figures 1, 2, 3).

Due to the severity of neurological symptoms and strong clinical suspicion of Wernicke encephalopathy, empiric high‐dose intravenous thiamine hydrochloride (500 mg three times daily for 5 days) was initiated to rapidly replenish thiamine stores. After clinical stabilization, oral supplementation with folic acid 5 mg once daily, calcium carbonate once daily, and iron–folic acid on alternate days was initiated. Once oral intake was tolerated, intravenous thiamine was transitioned to oral thiamine 200 mg three times daily for maintenance. Supportive therapy included short‐term lorazepam for sleep and agitation and low‐dose olanzapine for behavioral symptoms. Her mental status improved within 48 h, and nystagmus has also resolved. Lower limb weakness gradually improved over the following week. Timely recognition and management prevented progression to irreversible neurological injury, with normal fetal monitoring throughout the hospital admission.

At the time of discharge, the patient had shown significant improvement in gait and ocular symptoms, with residual mild deficits in recent memory and occasional confabulation. She was hemodynamically stable, tolerating oral intake, and was discharged on oral thiamine supplementation with close neurology and obstetric follow‐up. However, follow‐up at our center was not feasible as the patient was from a rural area. She was advised to continue follow‐up at a nearby hospital with neurological services available. The patient continued to improve after discharge, and the pregnancy subsequently progressed without complications. The patient delivered a single live healthy fetus weighing 2550 g via normal vaginal delivery with an APGAR score of 7/10 and 8/10 at 1 and 5 min, respectively, with no congenital anomalies. The immediate postpartum period was uneventful.

## 4 Discussion

A 34‐year‐old Secundigravida at 16th weeks gestation with no history of involuntary body movements or slurring of speech presented with multiple episodes of vomiting for 2 months and altered sensorium for 5 days associated with weight loss of about 5 kg and reduced oral intake. Subsequently, she developed gait ataxia, altered speech, and impaired recognition of family members. MRI revealed bilateral thalamic and periaqueductal hyperintensities, consistent with Wernicke's encephalopathy (WE).

Wernicke's encephalopathy (WE) is an acute neurological disorder caused by severe thiamine deficiency that manifests with a common range of clinical features, including a triad of global confusion, ophthalmoplegia, and ataxia [8]. In our case, the patient did not have a history of alcohol use and presented with only some features of the triad (confusion and nystagmus with mild gait ataxia).

The pathophysiology of WE in HG is the limited thiamine reserves in the body, which can be quickly depleted during pregnancy, particularly in cases of Hyperemesis Gravidarum (HG), where decreased intake, impaired absorption, and heightened metabolic requirements are prevalent [9]. In our patient, neurological manifestations developed after 7 weeks of severe vomiting, highlighting the rapid precipitation of Wernicke's Encephalopathy due to Hyperemesis Gravidarum. Most of the pregnancy‐related Wernicke's Encephalopathy occurs in the first or early second trimester [10], consistent with this case.

MRI Brain revealed T2/FLAIR high signal intensity in the bilateral medial thalami, periaqueductal gray matter, mammillary bodies, bilateral mesial temporal lobes, and insular cortex, suggestive of Wernicke's Encephalopathy. Typical lesions of Wernicke's Encephalopathy include symmetrical T2/FLAIR hyperintensities involving the medial thalami, mammillary bodies, and periaqueductal gray matter. There have also been reports of further involvement of atypical regions such as the insular cortex and mesial temporal lobes in pregnancy‐related and other nonalcoholic cases [11, 12]. The neuroimaging findings in our patient closely parallel those described in previously published cases of Wernicke's encephalopathy secondary to Hyperemesis Gravidarum, further supporting the diagnosis. The laboratory investigations of this patient revealed normocytic normochromic anemia along with marked hyperbilirubinemia and mildly elevated transaminases with normal alkaline phosphatase levels.

The management of WE is mainly thiamine supplementation. The European Federation of Neurological Societies (EFNS) recommends intravenous thiamine 200 mg three times daily, initiated before carbohydrate intake, with lower doses (100–200 mg once daily) often sufficient in nonalcoholic patients, whereas alcoholic patients may require higher dosing [13]. In our case, Wernicke's encephalopathy was diagnosed clinically, and intravenous thiamine was initiated promptly after suspicion, leading to significant neurological improvement and underscoring the importance of early empirical parenteral thiamine therapy.

Untreated Wernicke's Encephalopathy can result in Korsakoff Syndrome in the mother and growth restriction or preterm delivery in the fetus. The favorable maternal and fetal outcome in this case emphasizes the importance of timely recognition and treatment [14]. In our patient, who was at 16th weeks of gestation, mild ataxia, nystagmus, and altered sensorium were the only observed features of the classical triad. Despite this partial presentation, prompt administration of thiamine resulted in rapid recovery. Timely recognition and management led to significant maternal neurological improvement, and the patient subsequently delivered a healthy baby with no congenital defects, highlighting the importance of early intervention in pregnancy‐related Wernicke's Encephalopathy.

Our case highlights the importance of suspecting Wernicke's Encephalopathy in pregnant women with persistent vomiting accompanied by neurological symptoms such as confusion, ocular abnormalities, or ataxia. Wernicke's Encephalopathy is a rare but potentially life‐threatening complication, with the true incidence remaining uncertain as the available literature largely consists of case reports and small case series.

## 5 Conclusion

Wernicke's Encephalopathy is a rare but reversible neurological emergency in pregnancy, rarely precipitated by Hyperemesis Gravidarum. Persistent vomiting with neurological symptoms should prompt immediate thiamine administration. Early recognition and treatment can prevent permanent maternal complications and ensure favorable fetal outcomes.

### 5.1 Ethical Consideration

Written informed consent was obtained from the patient for publication of this case report and accompanying images.

#### 5.1.1 Limitation

1. This case has several limitations. Serum thiamine levels were not measured due to the unavailability of testing facilities.
2. Imaging was limited to MRI, and long‐term cognitive follow‐up beyond 6 months could not be performed.
3. Additionally, as this is a single case report, the findings may not be generalizable.
4. Furthermore, there are currently no standardized screening protocols or clear recommendations regarding empiric treatment of Wernicke's encephalopathy in patients with hyperemesis gravidarum, highlighting the need for further research to establish optimal screening and management strategies.

## Author Contributions

**Abhishek Mahato:** conceptualization, data curation, supervision, validation, writing – original draft. **Sameer Parajuli:** data curation, visualization, writing – original draft. **Bandana Ghimire:** writing – original draft, writing – review and editing. **Bishal Basnet:** data curation, validation, writing – original draft. **Bishal Sigdel:** data curation, visualization, writing – original draft. **Bishal Kumar Yadav:** data curation, validation, writing – original draft. **Sagar Kumar Jha:** writing – original draft, writing – review and editing.

## Funding

The authors have nothing to report.

## Ethics Statement

Our institution does not require ethical approval for reporting individual cases or case series.

## Consent

Written informed consent was obtained from the patient for the anonymized information to be published in this article.

## Conflicts of Interest

The authors declare no conflicts of interest.

## Provenance and Peer Review

Not commissioned, externally peer‐reviewed.

## Data Availability

All relevant data supporting the findings of this case report are included within the article. No additional datasets were generated. Additional anonymized information may be available from the corresponding author upon reasonable request, subject to ethical and privacy considerations.
